# Robot-based intervention may reduce delay in the production of intransitive gestures in Chinese-speaking preschoolers with autism spectrum disorder

**DOI:** 10.1186/s13229-018-0217-5

**Published:** 2018-05-24

**Authors:** Wing-Chee So, Miranda Kit-Yi Wong, Wan-Yi Lam, Chun-Ho Cheng, Jia-Hao Yang, Ying Huang, Phoebe Ng, Wai-Leung Wong, Chiu-Lok Ho, Kit-Ling Yeung, Cheuk-Chi Lee

**Affiliations:** Department of Educational Psychology, The Chinese University of Hong Kong, Hong Kong, Hong Kong, Special Administrative Region of China

**Keywords:** Gesture, Autism spectrum disorder, Robot-based intervention, Early childhood

## Abstract

**Background:**

Past studies have shown that robot-based intervention was effective in improving gestural use in children with autism spectrum disorders (ASD). The present study examined whether children with ASD could catch up to the level of gestural production found in age-matched children with typical development and whether they showed an increase in verbal imitation after the completion of robot-based training. We also explored the cognitive and motor skills associated with gestural learning.

**Methods:**

Children with ASD were randomly assigned to two groups. Four- to 6-year-old children with ASD in the intervention group (*N* = 15) received four 30-min robot-based gestural training sessions. In each session, a social robot, NAO, narrated five stories and gestured (e.g., both hands clapping for an awesome expression). Children with ASD were told to imitate the gestures during training. Age-matched children with ASD in the wait-list control group (*N* = 15) and age-matched children with typical development (*N* = 15) received the gestural training after the completion of research. Standardized pretests and posttests (both immediate and delayed) were administered to assess the accuracy and appropriateness of gestural production in both training and novel stories. Children’s language and communication abilities, gestural recognition skills, fine motor proficiencies, and attention skills were also examined.

**Results:**

Children with ASD in the intervention condition were more likely to produce accurate or appropriate intransitive gestures in training and novel stories than those in the wait-list control. The positive learning outcomes were maintained in the delayed posttests. The level of gestural production accuracy in children with ASD in the delayed posttest of novel stories was comparable to that in children with typical development, suggesting that children with ASD could catch up to the level of gestural production found in children with typical development. Children with ASD in the intervention condition were also more likely to produce verbal markers while gesturing than those in the wait-list control. Gestural recognition skills were found to significantly predict the learning of gestural production accuracy in the children with ASD, with such relation partially mediated via spontaneous imitation.

**Conclusions:**

Robot-based intervention may reduce the gestural delay in children with ASD in their early childhood.

## Background

Individuals with autism spectrum disorder (ASD) are characterized by impairments in social interaction and communication (Diagnostic and Statistical Manual of Mental Disorders, Fifth Edition, DSM-V; [1]). Approximately half of them do not attain fluent speech and a quarter of them do not have functional speech [25, 30, 59]. Therefore, alternative and augmentative communication (AAC) systems serve important means of their communication. Besides aided AAC including Picture Exchange Communication System (PECS) [8] and speech-generating devices (SGDs), unaided AAC, gesture, can supplement (i.e., augment) their existing speech or become their primary (i.e., alternative) method of expressive communication (see discussion of manual signs in [51]).

Of different kinds of gestures (e.g., body posture, facial expression, a motion of hand or head), the present study focused on hand gestures. Hand gestures are spontaneous hand movements produced for communication (e.g., waving a hand to say goodbye; swiping forehead with hand when feeling hot); we gesture when we talk. Previous research has found that young English-speaking children with ASD have delayed gestural development, in comparison to their age-matched children with typical development and those with developmental delay [9, 16, 45, 47, 79]. For example, young children with ASD have difficulties in producing “proto-declarative” pointing gestures (gestures that elicit joint attention and shared interests, e.g., a child points to a toy car in order to direct his or her mother’s attention to it) [4, 14, 77, 78]. They also produce fewer markers (gestures that carry culturally specific meaning for communication, e.g., the raised thumb for hitchhiking) [16, 45, 47, 79] and iconic gestures (gestures that depict the actions or attributes of the entities in question, e.g., both hands flapping for a bird or to indicate flying) than children with typical development [16, 45, 79]. The delay in gestural use is still evident in middle and late childhood. In a study conducted by So et al. [73], 6- to 12-year-old children with ASD were found to gesture less often and use fewer types of gestures, especially markers, in comparison to their age-matched children with typical development. These children were also found to have difficulty producing iconic gestures at specified locations to identify entities [69, 73]. The severity of social and communication impairments may influence the production of communicative gestures [71].

However, some studies have not found this delay in gestural use throughout childhood and adolescence in individuals with ASD. Attwood et al. [2] reported that young children with ASD, children with Down’s syndrome (DS), and children with typical development produce comparable numbers of markers. Similarly, Capps et al. [13] found that young children with ASD were as likely as children with developmental delay to enact activities with iconic gestures. de Marchena and Eigsti [20] have shown that there is no significant difference in overall gesture rate and in the number of different types of gestures produced between adolescents with ASD and their age-matched adolescents with typical development. Likewise, Medeiros and Winsler [50] reported that 7- to 18-year-old children with ASD and children with typical development produce similar numbers of iconic gestures and speech beats. Wong and So [82] even found that 6- to 12-year-old children with ASD produce more iconic gestures when narrating stories than their age-matched children with typical development.

While there are inconclusive results regarding the delay in gestural use in children and adolescents with ASD, Ham et al. [28] proposed that individuals with ASD may have selective delayed development of one type of gesture, namely, intransitive gestures (actions without objects but with symbolic meanings). These gestures convey socio-communicative intent (e.g., waving a hand to say goodbye, giving a thumbs-up for a great job, opening arms wide to welcome others). A case report by Ham et al. [28] found that an 11-year-old child with ASD has difficulties producing intransitive gestures. In their later work, they found that children with ASD had greater difficulties recognizing intransitive gestures than gestures that involve actions with objects [29]. Their results are in line with previous findings, in which young and school-aged children have specific difficulties producing markers [16, 45, 47, 73, 79]. Intervention studies designed to teach children with ASD the use of intransitive gestures are scarce. The present study aimed to teach children with ASD, specifically preschool children, intransitive gestures. Early intervention is the key for success in improving their communication skills and social competence before they enter mainstream primary schools. Additionally, it is easier to teach young children appropriate behaviors and new skills at the time when the brain is most easily developed.

In this study, we adopted a social robot as a teaching agent. Previous research has shown that individuals with ASD tend to have low levels of interest toward other humans and have a weaker understanding of the interpersonal world than of the object-related world (e.g., [36, 37, 40]). In addition to this, they find it challenging to pay attention to multiple cues during social interactions with humans [38]. Thus, they are not sensitive to other people’s behaviors [41]. Individuals with ASD are therefore more responsive and respond more quickly to feedback given by a technological object than by a human (e.g., [58, 83]).

Among different technological objects, they prefer robot-like toys to non-robotic toys and human beings [18, 61]. Based on the empathizing-systemizing theory [5], robots, unlike humans, operate within predictable and lawful systems and are thereby favored by children with ASD. Social robots have been widely used in therapy for individuals with ASD in the past decade (see reviews in [11, 24, 42]). They (1) offer human-like social cues; (2) can be programmed in such a way that information can be repeated in the same format; and (3) are predictable and controllable. Therefore, social robots can provide structured and clear information. They also respond to children with ASD according to predictable rules [7]. Additionally, these children do not need to consider socio-emotional expectations when interacting with robots [68], thereby reducing their social anxiety [52].

Recently, So and colleagues conducted a first multi-phase robot-based intervention study on gesture use for Chinese-speaking school-aged children with ASD [70]. School-aged children with ASD were taught to recognize, imitate, and produce 20 gestures of different types, demonstrated by the robot animation in three phases. Their results reported that children recognized and imitated more gestures, as well as producing them in appropriate social contexts after training. There were significant differences between the pretests and posttests across the three phases. The children also generalized their acquired gestural skills to a novel setting with a human researcher. In another study, they used a real social robot (as opposed to robot animation) to teach Chinese-speaking school-aged children with ASD to recognize and produce eight gestures that express feelings and needs (e.g., NOISY) [72] in two phases. Compared to the students in the wait-list control group who had not received training, students in the intervention group were more capable of recognizing and producing the eight gestures produced in the trained and non-trained scenarios. Significant differences between the pretests and posttests were found across the two phases. Even more promising, these students could recognize the same gestures produced by human experimenters. However, there was no strong evidence showing that the children in the intervention group could generalize the acquired gestural production skills to interactions with human experimenters.

At present, previous findings have shown that the gestural recognition and production of children with ASD improves after receiving robot-based training. These results are encouraging, as they provide an effective treatment of gestural communication for children with ASD in educational and clinical settings. Yet, it is *not* known whether or not children with ASD still have delayed gestural production, in comparison to their age-matched children with typical development, after the completion of robot-based training. The first objective of the present study addressed this issue. We investigated whether or not children with ASD could catch up to the level of gestural production found in age-matched children with typical development in both trained and novel situations. If so, children with ASD would produce the target gestures as accurately as their age-matched children with typical development, suggesting that our robot-based intervention could reduce gestural delay in children with ASD. Otherwise, the children with ASD would still gesture less accurately than their children with typical development even after the completion of the robot-based intervention, suggesting that our robot-based intervention could not reduce gestural delay in children with ASD.

The second objective of the present study would examine whether an improvement in gestural production skills, if any, would be associated with increases in verbal imitation in children with ASD, even though our intervention did not directly target verbal imitation. Previous research has shown that better imitation skills were associated with language gains, which were found immediately after imitation training as well as a few years later, in children with ASD [14, 75, 76]. These findings suggested that imitation skills may play a fundamental role in shaping language skills in children with ASD. Among different kinds of imitation, a study by Ingersoll and Lalonde [32] reported that gestural imitation training (as opposed to object imitation training) yielded a gain in language use. During the training phase in the present study, children with ASD were encouraged to imitate the social robot to produce intransitive gestures while listening to the narration. Based on previous findings, we expected that gestural imitation would trigger verbal imitative behaviors, such that a child would learn to produce the intransitive gestures (e.g., come over) while saying the accompanying words (“come over”). The integration of speech and gestures is crucial when one is narrating a story.

Besides investigating the effectiveness of the robot-based intervention on gestural use and verbal imitation, we also examined whether underlying cognitive and motor skills may predict gestural learning outcomes in children with ASD, including language and communication ability, gestural recognition, fine motor skills, and attention skills. These factors have not been sufficiently studied in the past gestural intervention research and are therefore measured in the present study. Among these factors, language and communication ability may be correlated to the production of gestures in early childhood. Previous research has shown that bilingual preschoolers used more gestures (iconic and beat) with their stronger language than with their weaker language [53, 54]. Other studies have shown that adult speakers use gestures (deictics) more often with their weaker language (e.g., [27, 55, 66]).

In addition to language and communication ability, the ability to identify the meanings of gestures (gestural recognition) may also be associated with gestural imitation or production skills. It was proposed that the mirror neuron system (MNS) plays a role in observational learning and imitation in children [62, 63]. Children with ASD may have dysfunction in the MNS, leading to the poor observation of gestures [57, 81]. Poor observation would then result in an impaired understanding of gestures, thereby causing difficulties in gestural imitation. As a result of this, the extent to which children with ASD comprehend gestures influences how well they can imitate the gestures. This proposal is in line with previous findings, which have shown that individuals with ASD imitate meaningful gestures more successfully than non-meaningful ones (e.g., [17, 80]).

Moreover, dysfunction in the MNS may result in motor deficits in children with ASD [15], which may in turn influence children’s ability to imitate and produce gestures. Motor deficits in autism can be subdivided into two main categories: (a) deficits in basic motor control and (b) difficulty with praxis performance. The latter is associated with the social, communicative, and behavioral impairments that are typical of autism [21, 22]. Recent research has shown that praxis skills are found to be correlated to the imitation of gestures [26], which requires the production of coordinated sequences of movements. Furthermore, attention is the key skill in learning, including gestural production. It is common for children with ASD to have attention impairments [19, 67], which may preclude them from developing effective learning strategies for producing gestures.

## Methods

### Participants

A total of 45 Chinese-speaking (Cantonese-speaking) participants aged 4 to 6 years old participated in this study. Of these, 30 had been diagnosed with autism or another autistic disorder when they were between the ages of 18 and 36 months (*M* = 30.27; SD = 8.17) by pediatricians at the Child Assessment Center for the Department of Health in Hong Kong. All the participants were attending various special care centers in Hong Kong. Their ASD diagnoses were further confirmed by clinical psychologists who administered Autism Diagnostic Observation Schedule (ADOS; [43]) and the Autism Diagnosis Interview-Revised (ADl-R; [44]) and by pediatricians from the Pamela Youde Child Assessment Center, Hong Kong, who followed the Diagnostic and Statistical Manual of Mental Disorders, Fifth Edition (DSM-V; [1]).

Participants with ASD were randomly assigned to two groups: an intervention group and a wait-list control group. Participants in the intervention group (*N* = 15, two females) received robot-based gestural training, while those in the wait-list control group (*N* = 15, one female) were trained after the completion of the research. The mean age of the participants in the intervention group was 5;10 (years; months) (SD = 0.83; range 4;2–6;12) and that of the wait-list control group was 5;8 (SD = 0.35; range 5;1–6;4).

The remaining 15 participants were age-matched and had not been diagnosed with ASD, i.e., participants with typical development (six females: *M* = 5;4; SD = 0.67; range 4;5–6;4). These children did not receive robot-based gestural training. There was no significant difference in age between the participants with ASD and those with typical development, Mann-Whitney (*U*) = 147.50, *p* < .11. None of the participants with typical development had a family history of ASD or other diagnosed developmental disorders or impairments. Neither the participants with typical development nor the participants with ASD had any history of traumatic brain injuries, birth-related injuries, or disorders involving seizures. All of the procedures were approved by the institutional review board of the first author’s university, in compliance with the Declaration of Helsinki (Reference no. 14600817). We obtained parents’ informed consent prior to the study. The participants also gave their assent to participate in this study.

At the beginning of the experiment, the participants with ASD and the participants with typical development had their language and communication abilities, fine motor skills, attention skills, and gestural recognition assessed, as these skills could influence their gestural learning. The order of these assessments was counterbalanced across the participants. Table 1 shows the descriptive statistics of the performance in each assessment for both groups of participants.

**Table 1:** Descriptive statistics of the participants’ performance in the PEP3, SCQ, BOT, ANT, and gestural recognition task

Groups	Descriptive statistics	Chronological age	Language and communication developmental age assessed by PEP-3	Standardized score in BOT	Proportion of accurate trials in ANT	Proportion of accurate trials in gestural recognition
Participants with ASD in the wait-list control	*M*	5.81	4.44	85.33	0.60	0.65
	SD	0.83	0.89	12.38	0.28	0.20
	Min	4.16	3.19	62.00	0.19	0.29
	Max	6.96	6.22	105.00	0.97	1.00
Participants with ASD in the intervention condition	*M*	5.65	4.95	105.07	0.74	0.69
	SD	0.35	0.44	20.15	0.21	0.25
	Min	5.06	4.53	49.00	0.31	0.14
	Max	6.28	5.83	130.00	1.00	1.00
Participants with typical development	*M*	5.31	5.41	112.13	0.83	0.85
	SD	0.67	0.46	14.19	0.10	0.11
	Min	4.43	4.72	86.00	0.69	0.64
	Max	6.35	6.22	128.00	1.00	1.00

*PEP3* Psychoeducational Profile-Third Edition [65], *BOT* Bruininks-Oseretsky Test of Motor Proficiency, Second Edition (BOT™-2; [10]), Attention Network Task (ANT; [64])

The participants’ language and communication abilities were measured by the Psychoeducational Profile, Third Edition (PEP-3; [65]). The PEP-3 examines the skills and behaviors of young children (aged from 6 months to 7 years) with autism and communication disabilities and charts their uneven and idiosyncratic development, emerging skills, and autistic behaviors. It has 10 subtests, which yield three composite scores in the following three aspects: language and communication, motor, and maladaptive behaviors. Our study focused on the language and communication composite, which measures a participant’s ability to speak, listen, read, and write, with a higher score indicating better language and communication skills. We reported here the language and communication developmental ages that were converted from the raw and standardized scores based on the norming references published in the PEP-3 administration manual. Unsurprisingly, the participants with typical development had better language and communication abilities than the participants with ASD, *U* = 55.50, *p* < .001.

The participants’ motor skills were assessed by the Bruininks-Oseretsky Test of Motor Proficiency, Second Edition (BOT™-2; [10]). It assesses the fine motor as well as the gross motor proficiencies of children, ranging from those who are typically developing to those with mild to moderate motor control problems. It has eight subtests, including Fine Motor Precision, Fine Motor Integration, Manual Dexterity, Bilateral Coordination, Balance, Running Speed and Agility, Upper-Limb Coordination, and Strength, which yield six composite scores, including Fine Manual Control, Manual Coordination, Body Coordination, Strength and Agility, Gross Motor Composite, and Fine Motor Composite. We only report the Fine Motor Composite standardized score here, as fine motor skills are related to the production of the intransitive gestures taught in the present study. This composite consists of the following subtests: Fine Motor Precision, Fine Motor Integration, Manual Dexterity, and Upper-Limb Coordination. The participants with typical development received a higher fine motor composite standardized score than the participants with ASD, *U* = 132.00, *p* < .03.

The participants’ attention skills were measured by the Attention Network Test (ANT) [23, 64]. This test lasts for half an hour and provides a nonverbal measure of the efficiency of the attentional networks involved in alerting, orienting, and executive attention across all ages in both typical and atypical populations. We focused on executive attention, which requires the participant to respond by pressing two keys indicating the direction (left or right) of a central arrow surrounded by congruent, incongruent, or neutral flankers. Thus, it evaluates one’s ability to focus on the relevant stimulus while ignoring the distracting but irrelevant stimuli. For each participant, we averaged the proportions of the trials to which he or she responded correctly in the congruent and incongruent conditions. There was no significant difference between the groups, *U* = 162.00, *p* < .13.

The participants’ gestural recognition skills were measured by their ability to identify the meanings of intransitive gestures demonstrated by an experimenter [70, 72]. We videotaped an experimenter producing the 14 intransitive gestures that were taught in the present study. Each time, a child was shown a gesture (e.g., both hands clapping) and was asked to choose one of three options that best identified its meaning (e.g., HELLO, AWESOME, WELCOME). We reported the proportion of trials each child was able to recognize the meanings of gestures. The proportion of accurate trials in the recognition test was higher in the participants with typical development than in those with ASD, *U* = 114.00, *p* < .007.

### Stimuli

#### Target gestures

A total of 14 intransitive gestures that are commonly used in daily life were taught in this intervention program. The findings of a study by Cabibihan, So, and Pramanik [12] showed that these gestures are easily recognized by speakers in the Chinese society.

#### Social robot

**Fig. 1:**
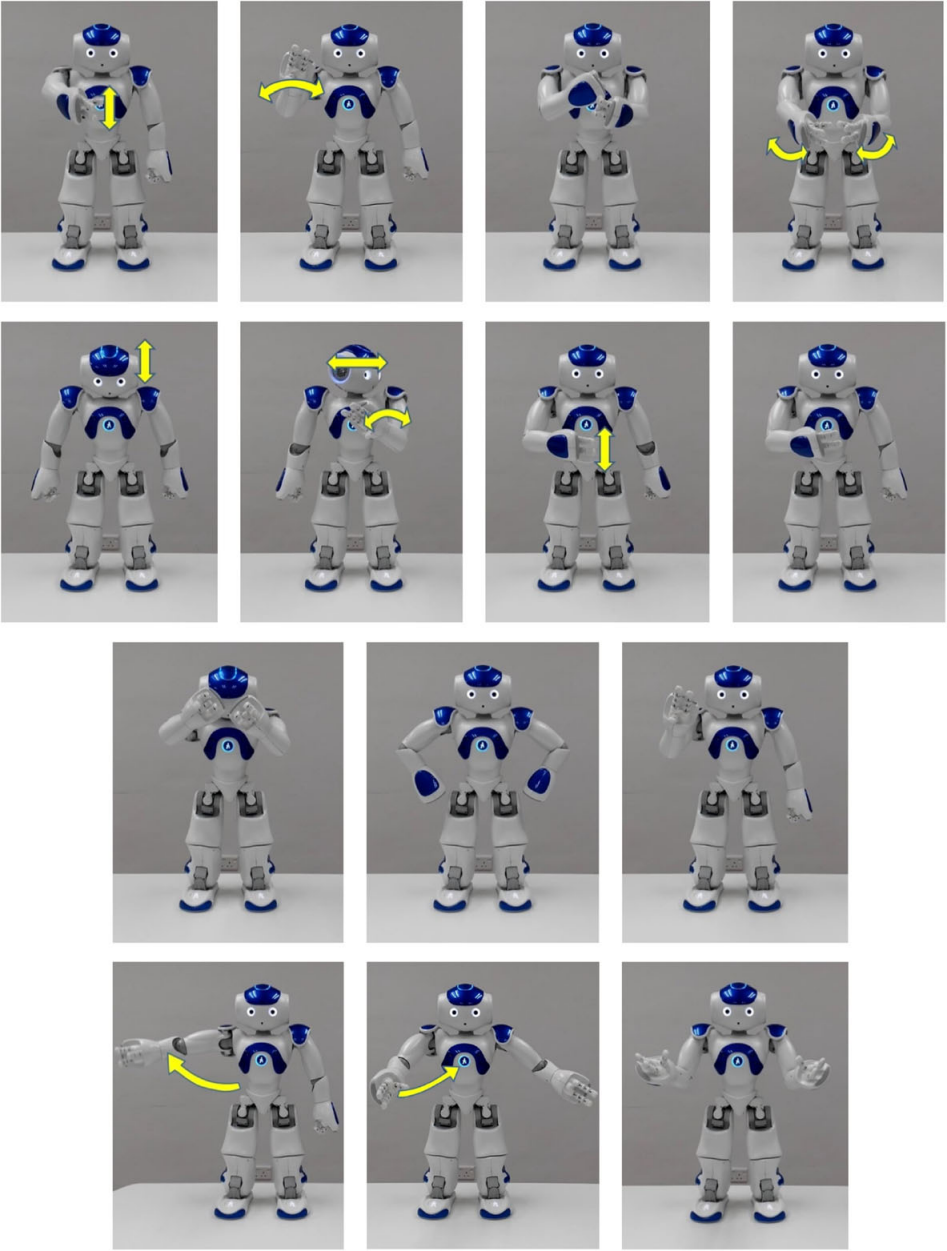
Gestures performed by the NAO robot. From the upper left corner, from left to right, the following gestures are (first row) hello, bye, wrong, and awesome; (second row) yes, not allowed, hungry, and myself; (third row) annoyed, angry, and wait; and (fourth row) welcome, come, and where?

NAO (Aldebaran Robotics Company) was programmed to produce the 14 gestures (see Fig. 1). This robot has been widely used in autism therapy. It is 50 cm tall and anthropomorphic. It was deployed in the present study because it might facilitate children with ASD to generalize the acquired imitation and social skills to human-to-human interactions [11]. Besides, unlike other robots, NAO robot can produce a wide range of gestures. The NAO robot contains 25 degrees of freedom (DOF) from 15 joints and actuators. Our gestures were accomplished by 14 DOF from nine joints and actuators. Each of the gestural movements required two DOF in the neck, two DOF in each shoulder, two DOF on each elbow, and one DOF on each wrist. Each gesture lasted for 3–4 s.

In order to ensure that the gestures produced by the NAO robot could be recognized by the participants, we asked the participants with typical development in this study to identify the meanings of the gestures demonstrated by the robot (the robot gesture recognition task). The procedure was similar to those in the aforementioned gestural recognition task (the human gesture recognition task). The order of the human gesture recognition task and robot gesture recognition task was counterbalanced across participants. In the robot gesture recognition task, the participant was presented with one video clip each time, which showed the robot producing a gesture, and he or she was asked to choose one of three options that best described its meaning. We entered the participants’ responses in both recognition tasks in the reliability analysis and found that the Cronbach’s alpha was .83, suggesting that the recognition of human and robot gestures was highly correlated.

#### Stories

In the training sessions, the robot gestured while narrating a set of five different stories (S1, training stories). For example, one of the S1 stories was as follows: “One day, a friend visited me and she was hungry. She wanted to dine out. I asked her where we should go for lunch. She suggested eating hamburgers. I said, ‘awesome’.” Each story in S1 contained five sentences and two to three different gestures. Each gesture only appeared once across all stories. Five different stories thereby covered all 14 gestures. This set of stories, S1, was used for training and assessment in the standardized pretests and posttests. Another set of stories (S2, non-training stories) contained five stories that were different from those in S1. S2 was presented during the assessment (but not training) sessions in order to examine the generalization effects of the intervention in the novel context.

The stories in S1 were told by a female Cantonese speaker and her narration was recorded as audio clips. To make the recordings sound like the speech produced by a robot, robotic effects were added and the speech rate was reduced using an audio editor (Audacity, v. 2.1.0, the Audacity Team, US state). A total of five audio clips were made, each containing one story, and these were imported to NAO. The mean length of each clip was 32.2 s (ranging from 25 to 42 s). NAO then played the audio clips and gestured during the narration. The gesture and its accompanying speech started at the same time. Thus, the participants with ASD in the intervention condition watched the gestures while listening to the stories. For example, in the aforementioned story, NAO said and gestured: “I asked her where (WHERE: two arms open wide with palms facing up) we should go for lunch”. In addition to the verbal narration, the background images of the stories (e.g., a living room) were visually displayed on the laptop screen, which was placed next to NAO. One picture was shown for each story.

### Procedures

**Fig. 2:**
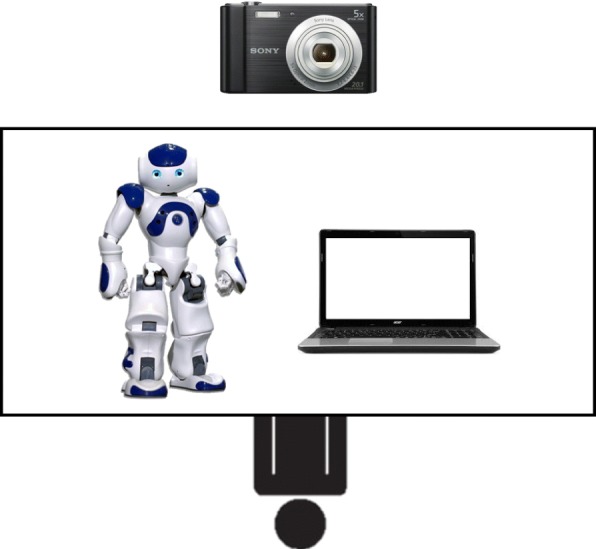
The experimental setting of training in the intervention condition

The experiment was conducted in the treatment rooms at various special care centers in Hong Kong for participants with ASD and in kindergarten classrooms for the participants with typical development. The treatment rooms and classrooms were often used by the children for school activities. Each time, the room was equipped with a robot, NAO, a laptop, and a camera in front of the child (see Fig. 2). The camera videotaped the hand movements the child produced in the sessions.

The intervention program lasted for 9 weeks. It consisted of two pretests (one for each set of stories: S1 and S2), four training sessions for S1 (with two 30-min sessions per week), two immediate posttests (which were the same as the pretests), and the same follow-up posttests after 2 weeks. The posttest for S1 assessed the training effects, while that for S2 assessed the generalization effects.

For each training or assessment session, the participants were accompanied by a teacher. The training and assessment sessions were administered by a researcher, who was either the assistant or one of the authors. A small reward by way of positive reinforcement (snacks or access to toys) was offered by the teacher at the end of each pretest, posttest, and training session. All the sessions were videotaped. Each session lasted for approximately 30 min. Details of the intervention program are provided below.

Each pretest assessed the production of 14 intransitive gestures in different stories (S1 and S2) in the participants with ASD (both in the intervention and the wait-list control groups) and the participants with typical development. At the beginning of the pretest, the human researcher first greeted the participant then gave instructions. The researcher then asked the participant whether he or she understood the instructions. If the participant indicated that he or she did not understand the instructions, the researcher would repeat the instructions. Otherwise, the human researcher proceeded to narrate a story, with the corresponding pictures sequentially displayed on the laptop screen. After narrating a story once, the researcher repeated the sentences (e.g., “One day, a friend visited me and she was hungry.”), one at a time. After finishing each sentence, the researcher asked the participant to demonstrate the corresponding gesture (e.g., “What are the hand movements for expressing the feeling of being hungry?”). The participant was given 10 s to respond. The researcher prompted the participant if he or she gave no response and gave the participant another 10 s to respond. Upon receiving the participant’s response, the researcher then judged the accuracy of the gesture produced (based on the four parameters which were further elaborated in the session below) and provided feedback (correct or incorrect). The researcher then proceeded to the next sentence. After the completion of one story, the researcher moved on to the next story. The two pretests were completed after the participant had been asked to demonstrate the individual gestures in the stories in S1 and S2. Each pretest lasted for approximately 30 min. A small reward by way of positive reinforcement was provided after the pretest.

After the pretest, the participant with ASD in the intervention group proceeded to training, in which the robot narrated the stories (each one twice) while producing appropriate gestures in S1. Each time, the participant was asked to imitate the gesture. The training was completed after the five stories had been presented and the corresponding gestures had been imitated by the child in S1. The participants with ASD in the wait-list control group and the participants with typical development watched educational videos that were not relevant to gestural training (e.g., videos about animals) for 30 min.

All participants then took the posttests immediately after the training and the delayed posttests 2 weeks after the training. The human researcher administering the posttests was the same as in the pretests. The procedures in both posttests were the same as those in the pretests. All participants were able to pay attention during the training and assessments. A short break was given to the participants if requested. None of them were absent from the sessions.

### Coding and scoring

We had a research assistant, who did not know the objectives of the study and was unaware of the research questions of the present study, watch the videos of the participants and counted the number of trials in which they produced the gestures correctly, according to four parameters [74]: use of hand/hands (e.g., placing the right/left hand against the head vs. using both hands), hand-shape (e.g., open palm vs. curled palm vs. fist), direction of movement (e.g., head nods vs. head shakes; moving the hand from left to right vs. moving it up and down), and placement (e.g., hand placed at the head vs. at the chest). The following gestures were considered incorrect: using only the left hand to produce the WHERE gesture (reason—incorrect use of hands); making a fist when producing the WAIT gesture (reason—incorrect hand shape); moving the right hand downward when producing the NOT ALLOWED gesture (reason—incorrect direction of movement); and covering the face when producing the MYSELF gesture (reason—incorrect placement).

For the gestures that did not follow the four parameters, we further coded whether these gestures were still appropriate. A gesture was considered appropriate if it conveyed the target meaning. For example, two index fingers formed a cross when producing the NOT ALLOWED gesture; right hand moved in a circle in the lower chest when producing the HUNGRY gesture; both hands raised up when producing the gesture AWESOME; and both hands formed a T-shape when producing the gesture WAIT.

Finally, we counted the number of trials in which the participants produced verbal imitation when they gestured (either accurately or appropriately). Verbal imitation was defined as the imitation of the robot’s verbal marker during gestural production (e.g., saying, “Awesome” while producing the AWESOME gesture).

We then train a second coder, who also did not know the objectives of the study and was unaware of the research questions of the present study, to code gestures. She watched 20% of the videos. The inter-observer agreement in an evaluation of the accuracy of gesture production was .93 (*N* = 756; Cohen’s kappa = .90, *p* < .001), that of appropriateness of gesture production was .96 (*N* = 580, Cohen’s kappa = .92, *p* < .001), and that of verbal imitation was .92 (*N* = 1336, Cohen’s kappa = .90, *p* < .001).

## Results

We first report the proportion of trials in which the participants with ASD in the intervention condition and those in the wait-list control condition and the participants with typical development accurately produced gestures in the pretests and immediate and delayed posttests. All children spontaneously produced the intransitive gestures when instructed or prompted by the human researcher.

Table 2 shows the correlations among chronological age, language and communication developmental age, fine motor skills, attention skills, gestural recognition skills, and gestural production performance in the pretests and posttests (the data in both S1 and S2 were collapsed). There were significant correlations between fine motor skills, attention skills, gestural recognition skills, and gestural production performance in the pretests and/or posttests. Language and communication developmental age was marginally correlated to the gestural production performance in the pretest (*p* < .07).

**Table 2:** Correlations among chronological age, language and developmental ability, gestural recognition, fine motor skills, attention skills, and gestural production accuracy in pretests and immediate and delayed posttests

	Chronological age	Language and developmental ability	Gestural recognition	Fine motor skill	Attention skill	Gestural production accuracy in pretests	Gestural production accuracy in immediate posttests	Gestural production in delayed posttests
Chronological age	–							
Language and developmental ability	.65**	–						
Gestural recognition	0.02	.33*	–					
Fine motor skill	.34*	.57**	0.23	–				
Attention skill	0.10	0.25	.66**	.40*	–			
Gestural production accuracy in pretests	0.05	0.28	.63**	0.22	.64**	–		
Gestural production accuracy in immediate posttests	0.04	0.23	.33*	.44**	.36*	0.20	–	
Gestural production in delayed posttests	0.04	0.19	.41**	.33*	.50**	.36*	.68**	–

**p < .001, *p < .05

**Fig. 3:**
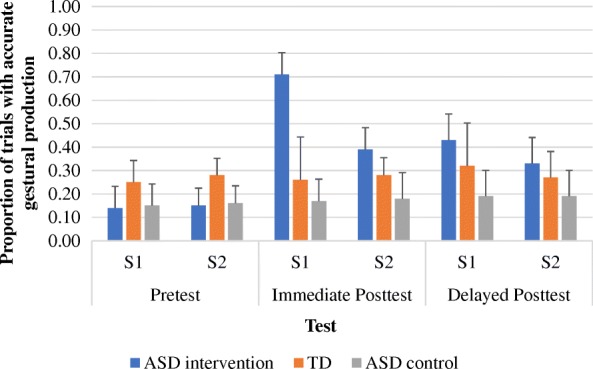
The proportion of accurate trials in the pretests and immediate and delayed posttests in the ASD children (intervention and wait-list control conditions) and children with typical development (TD) in S1 and S2 narratives

Figure 3 shows the proportion of accurate trials in the gestural production pretests and immediate and delayed posttests in the participants with ASD (intervention and wait-list conditions) and in the participants with typical development. Repeated measures ANOVA, with group (participants with ASD in the intervention condition, participants with ASD in the wait-list control, participants with typical development) as the between subject factor, time (pretest, immediate posttest, delayed posttest) and story (S1, S2) as the within subject factors, and language and communication developmental age, BOT standardized score, and the proportions of correct trials in the ANT and gestural recognition tasks as covariates, was conducted. We found significant effects for group, *F*(2, 38) = 24.44, *p* < .001, *ηp*^2^ = .56, time × group interaction, *F*(4, 76) = 22.39, *p* < .001, *ηp*^2^ = .54, story × group interaction, *F*(2, 38) = 30.50, *p* < .001, *ηp*^2^ = .62, and three-way interaction, *F*(4, 76) = 10.12, *p* < .001, *ηp*^2^ = .35. All the other main and interaction effects were not significant.

We further explored the three-way interaction by running two separate repeated measures ANOVAs for S1 and S2, respectively, after controlling for language and communication ability, fine motor skills, attention skills, and gestural recognition skills. With regard to S1, which was a set of stories presented during training, we found significant effects for group, *F*(2, 38) = 37.04, *p* < .001, *ηp*^2^ = .66, and time × group interaction effects, *F*(4, 76) = 25.16, *p* < .001, *ηp*^2^ = .57. Post hoc Tukey HSD have shown that the proportion of accurate trials in the pretest with the participants with typical development was greater than that with the participants with ASD in the intervention condition, *p* < .04, and those in the wait-list control condition, *p* < .05. Opposite patterns were found in the immediate posttest. The proportion of accurate trials with the participants with ASD in the intervention condition was greater than that for the participants with typical development, *p* < .001, and for the participants with ASD in the wait-list control condition, *p* < .001. Marginal significance was found between the participants with typical development and the participants with ASD in the wait-list control condition, *p* < .07. Similar findings were shown for the delayed posttest. The proportion of accurate trials with the participants with ASD in the intervention condition was greater than that for the participants with typical development, *p* < .03, and for the participants with ASD in the wait-list control condition, *p* < .001. Marginal significance was found between the participants with typical development and the participants with ASD in the wait-list control condition, *p* < .08.

Regarding S2, which was a set of stories that had not yet been taught, we found significant effects for group, *F*(2, 38) = 7.24, *p* < .002, *η*^2^ = .28, and time × group interaction effects, *F*(4, 76) = 4.47, *p* < .003, *ηp*^2^ = .19. Post hoc Tukey HSD have shown that, similar to S1, the proportion of accurate trials in the pretest with the participants with typical development was greater than that for the participants with ASD in the intervention condition, *p* < .05, and that in the wait-list control condition, *p* < .03. Different from S1, the proportion of accurate trials in the immediate posttest with the participants with ASD in the intervention condition was comparable to that in the participants with typical development, *p* < .27, but greater than that in the participants with ASD in the wait-list control condition, *p* < .001. Significant difference was found between the participants with typical development and the participants with ASD in the wait-list control condition, *p* < .04. Likewise, the proportion of accurate trials in the delayed posttest in the participants with ASD in the intervention condition was comparable to that in the participants with typical development, *p* < .16, but greater than that in the participants with ASD in the wait-list control condition, *p* < .005. Marginal significance was found between the participants with typical development and the participants with ASD in the wait-list control condition, *p* < .06. Overall, the participants with ASD in the intervention condition were able to produce gestures as accurately as the participants with typical development in the non-training stories in the delayed posttest, suggesting that robot-based gestural intervention may reduce gestural production delay.

Interestingly, the proportions of gestures accurately produced in the participants with typical development were relatively low (below 30% for most of the assessments across all time points). Note that we coded the gestural production accuracy according to the four parameters (use of hands, hand shape, directionality of movement, and placement). It was possible that these participants produced appropriate gestures even though they did not follow the four parameters. In other words, their gestures might still convey the target meaning. We thus looked at their performance of gestural production by investigating their proportions of trials in which the gestures were accurately produced according to the four parameters as well as the proportions of trials in which the gestures were appropriate. Both proportions were then summed up.

**Fig. 4:**
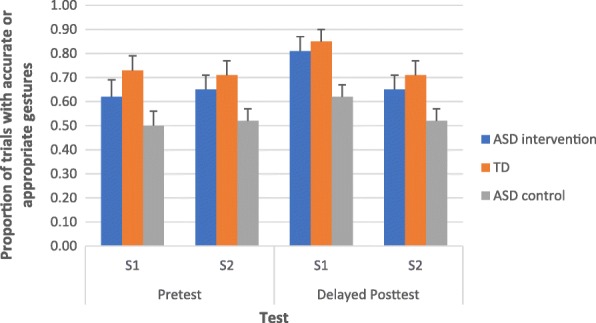
Proportion of trials in which the participants with ASD (intervention and wait-list conditions) and the participants with typical development produced gestures accurately or produce appropriate gestureᅟ

In this analysis, we only looked at gestural production in the pretests and delayed posttest. Figure 4 shows the proportion of trials in which the participants with ASD (intervention and wait-list conditions) and the participants with typical development produced gestures accurately or produce appropriate gestures. Repeated measures ANOVA, with group (participants with ASD in the intervention condition, participants with ASD in the wait-list control, participants with typical development) as the between subject factor, time (pretest and delayed posttest) and story (S1 and S2) as the within subject factors, and language and communication developmental age and gestural recognition tasks as covariates, was conducted. We found a significant effect for group, *F*(2, 38) = 3.74, *p* < .01, *ηp*^2^ = .25. All the other main and interaction effects were not significant. Bonferroni pairwise comparisons have shown that the proportion of trials with appropriate or accurate gestural production in the participants with typical development was higher than in those with ASD in the wait-list condition, *p* < .005. However, there was no difference between the participants with typical development and those with ASD in the intervention condition, *p* < .72. This result supported our previous results. The participants with ASD in the intervention condition were able to produce gestures as accurately or appropriately as the participants with typical development. Additionally, the proportions of trials with appropriate or accurate gestural production in the participants with ASD in the intervention condition were higher than in those with ASD in the wait-list condition, *p* < .03, suggesting that the robot-based intervention program was effective in promoting the use of appropriate or accurate gestures.

**Fig. 5:**
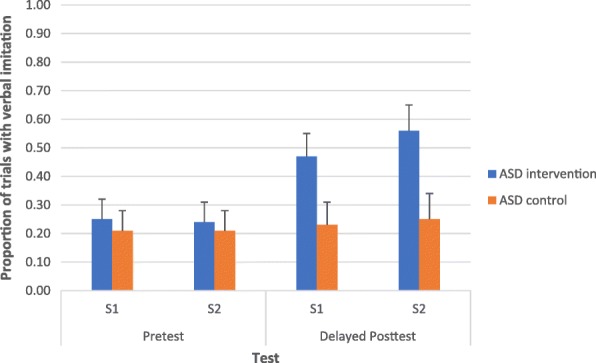
Proportion of trials in the gestural production pretests and delayed posttests in which the participants with ASD (intervention and wait-list conditions) used verbal imitation

Next, we investigated whether the participants with ASD in the intervention condition were more likely to use verbal imitation after learning target gestures. In this analysis, we compared the proportions of trials in which the participants with ASD in the intervention and wait-list control conditions used verbal imitation while producing appropriate or accurate gestures. Figure 5 shows the proportion of trials in the gestural production pretests and delayed posttests in which the participants with ASD (intervention and wait-list conditions) used verbal imitation. Repeated measures ANOVA, with group (participants with ASD in the intervention condition, participants with ASD in the wait-list control) as the between subject factor, time (pretest and delayed posttest) and story (S1 and S2) as the within subject factors, and language and communication developmental age and gestural recognition tasks as covariates, was conducted. All the main effects were not significant. Time × group interaction was significant, *F*(1, 26) = 4.56, *p* < .04, *ηp*^2^ = .15. All the other interaction effects were not significant. We further explored the time × group interaction by running two separate repeated measures ANOVAs for the participants with ASD in the intervention condition and those in the wait-list control condition, respectively, after controlling for language and communication ability and gestural recognition skills. For the participants in the intervention condition, we found a significant effect for time, *F*(1, 12) = 7.87, *p* < .02, *ηp*^2^ = .40. All the other main and interaction effects were not significant. Bonferroni pairwise comparisons have shown that the proportions of trials with verbal imitation in the delayed posttests were higher than in the pretests, *p* < .005. This finding was not reported in the participants in the wait-list condition. All the main and interaction effects were not significant. These results suggested that the robot-based intervention improved the gestural production accuracy and verbal imitation in the participants with ASD in the intervention condition.

Finally, we examined whether or not language and communication ability, fine motor skills, attention skills, and gestural recognition skills would influence gestural learning in the participants with ASD in the intervention condition. In order to address this issue, we focused on their accuracy of gestural production in the delayed posttests while controlling for their performance in the pretests. We ran a generalized linear mixed model analysis with individual gestures and subjects considered as the random effects, language and communication ability, fine motor skills, attention skills, gestural recognition skills, and performance in the pretests as the fixed effects, and the participants’ performance in the delayed posttest as the binomial dependent variable. The statistical analysis tools *R* [60] and lme4 [6] were used to perform the analyses. We collapsed the data in S1 and S2 in this analysis. We found that only gestural recognition was a significant predictor of gestural learning, *β* = .64, SE = .32, *p* < .04. Other factors were non-significant: language and communication ability, *β* = .05, *SE* = .01, *p* < .21; fine motor skills, *β* = .01, *SE* = .09, *p* < .13; and attention skills, *β* = .87, *SE* = .82, *p* < .29. These results suggest that a fundamental understanding of gestural meaning influences the learning of gestural production.

We ran a further analysis to explore how gestural recognition aids the learning of gestural production. Previous research has shown that imitating meaningful actions (from the perspectives of children with ASD) increases children’s natural motivation to complete the actions [31]. Previous findings have also shown that individuals with ASD imitate meaningful gestures better than non-meaningful ones (e.g., [17, 80]). Based on these views, recognition of gestures might lead to spontaneous imitation during training, which in turn would promote gestural production. Therefore, we watched the videos of the training sessions and recorded whether or not the participants with ASD in the intervention condition spontaneously imitated the gestures produced by NAO.

We expected spontaneous imitation during training to act as a mediator, which would influence the effects of gestural recognition skills on gestural learning among the participants with ASD in the intervention group. Therefore, a series of linear regression analyses were conducted to investigate how gestural recognition skills predicted gestural learning via the mediation of spontaneous imitation among participants with ASD in the intervention group. Gestural learning was measured by the residual scores of gestural production performance in the delayed posttest, after controlling for the effects of gestural production performance in the pretest.

We followed four steps in establishing mediation using regression [3, 33, 35]. Table 3 shows the statistics of the four regression analyses. In the first regression analysis, we found a significant effect of gestural recognition skills on gestural learning. In the second regression analysis, we found a significant effect of gestural recognition skills on spontaneous imitation. In the third regression analysis, we found a significant effect of spontaneous imitation on gestural learning. In the fourth regression analysis, gestural recognition skills still significantly predicted gestural learning after controlling for spontaneous imitation. These findings suggest that the effect of gestural recognition skills on gestural learning was partially mediated by spontaneous imitation during training.

**Table 3:** Statistics of four regression analyses showing the effects of gestural recognition skills on gestural learning via the mediation of spontaneous imitation among the children with ASD in the intervention group

Regression analyses	*β*	*t*	*R* ^2^	Δ*R*^2^	*F*
Step 1 (*N* = 414)					
DV: gestural learning					
IV: gestural recognition skills	.15	3.01**	.02	.02**	9.08**
Step 2 (*N* = 402)					
DV: spontaneous imitation					
IV: gestural recognition skills	.22	4.55***	.05	.05***	20.70
Step 3 (*N* = 402)					
DV: gestural learning					
IV: spontaneous imitation	.12	2.44*	.02	.02*	5.97*
Step 4 (*N* = 402)					
DV: gestural learning					
Block 1 IV: gestural recognition skills	.15	2.94**	.02	.02**	8.65**
Block 2 IVs: gestural recognition skills	.13	2.47*	.03	.01^†^	6.06**
Spontaneous imitation	.09	1.85^†^			

Note: ^†^*p* < .1; *p < .05; **p < .01; ***p < .001

## Discussion

To summarize, participants with ASD who received robot-based gestural training produced intransitive gestures more accurately in training stories than those who did not receive training. Similar patterns were found in non-training stories, suggesting that the acquired gestural production skills could be generalized to novel stories. Additionally, the positive learning outcomes were maintained for 2 weeks when no training was provided. Even more promising, the level of gestural production accuracy in participants with ASD in the delayed posttest of non-training stories was comparable to that in participants with typical development, suggesting that participants with ASD could catch up to the level of gestural production found in participants with typical development. This finding was consistent when we also considered appropriateness of gestures. Additionally, these participants with ASD were more likely to imitate the verbal markers paired with the taught intransitive gestures in the delayed posttests of both stories than in the pretests. Gestural recognition skills were found to significantly predict the learning of gestural production in the participants with ASD, with this relation being partially mediated by spontaneous imitation.

Previous findings have shown that robot-based gestural training is effective in teaching school-aged children with ASD [70, 72]. The present study represents significant steps forward in the development and therapeutic use of robot-based intervention. First, our results document that this intervention protocol works in preschool children, thus providing an effective early intervention for nonverbal communication skills. Additionally, the findings show that robot-based intervention may reduce the delay in gestural production in participants with ASD. Previous intervention studies have not included age-matched children with typical development [70, 72], thereby leaving open the question of whether or not participants with ASD who receive robot-based gestural training could catch up to the level of gestural production found in their age-matched participants with typical development peers. We found that the participants with ASD in the intervention condition had comparable performances to the participants with typical development in the immediate as well as in the delayed posttests in regard to non-training stories. This finding is intriguing given that, in comparison to their age-matched participants with typical development, participants with ASD have poorer language and communication abilities and fine motor proficiencies, which might influence gestural learning (see our discussion below). It also suggests that the delay in gestural production found in early childhood can be prevented by 2 weeks of robot-based gestural training.

It is interesting to observe that the participants with ASD in the intervention condition significantly outperformed the participants with typical development in the immediate posttest of the training stories. Specifically, their proportion of trials with accurate gestures increased from .15 in the pretest to .70 in the immediate posttest (while that of the participants with typical development remained at approximately .25 in both pre- and posttests). This result may be explained by the fact that the target intransitive gestures were visually presented to the participants with ASD in the intervention condition and individuals with ASD in general process information using a visually oriented approach [39]. Previous research has also shown that individuals with ASD may have superior short-term visual memory (e.g., [34, 56]). As a result of this, participants with ASD might benefit from the visual presentation of gestures and efficiently learn and memorize the gestures. Yet, their level of accuracy of gestural production declined in the delayed posttest of the same stories (.41), although it was still significantly higher than that of the participants with typical development. We did not observe superiority in the gestural production in the immediate and delayed posttests in non-training stories in the participants with ASD in the intervention condition, in comparison to the participants with typical development. This further strengthens our argument that short-term visual memory plays a significant role in learning and memorizing gestures incorporated in training stories. Despite this, the participants with ASD in the intervention condition could still generalize the acquired gestural production skills to the non-training stories, such that their accuracy of gestural production was greater than that of the participants with ASD in the wait-list control condition and was on par with that of the participants with typical development.

We also observed that the proportions of gestures accurately produced in the participants with typical development were below 30% for most of the assessments across all time points. It might be attributed to the fact that we coded the gestural production accuracy according to the four parameters (use of hands, hand shape, directionality of movement, and placement; [74]). Gestures do not contain standardized forms [48], and different forms of gestures may convey the same meaning (e.g., both hands forming a T-shape or right/left palm facing outward can request somebody else to stop from moving). Thus, the gestures produced by the participants should still be considered appropriate if they could convey the target meanings. After taking appropriateness of gestures into account, the proportions of accurate or appropriate gestures reached 70% or above in the participants with typical development across all time points. On the other hand, these proportions were comparable to those of children with ASD in the intervention condition. Taken together, the participants with ASD in the intervention condition were as capable as the participants with typical development in producing accurate gestures (i.e., those demonstrated by the robot) and appropriate gestures (i.e., those not demonstrated by the robot but were appropriate in conveying the intended meanings). In future studies, we should code the gestures in terms of their accuracy based on the four parameters as well as their appropriateness.

Previous research has shown that gestural imitation training can enhance language use [32]. In line with these findings, our study reported that participants with ASD in the intervention condition were more likely to produce the verbal markers that were co-occurring with the taught gestures in the delayed posttests than in the pretests. Such result was not found in the participants in the wait-list control condition. This was possibly because the demonstration of intransitive gestures by the robot drew the attention of the participants with ASD in the intervention condition to the verbal markers. Therefore, they noticed the gestures and verbal markers as a joint communicative art [48, 49] and produced the verbal markers while gesturing in the assessment. In this sense, gestural imitation would trigger verbal imitative behaviors. Teaching children with ASD who are non-verbal or having relatively low verbal abilities gesture would facilitate their speech production. Future studies on gestural training in children with ASD should evaluate the learning outcomes on gestural production as well as the development of speech and gesture integration.

The other major finding in the present study was that the learning of gestural production is influenced by gestural recognition skills. After controlling for the gestural production performance in the pretests of both training and non-training stories, the proportion of trials in which the participants with ASD recognized gestures significantly predicted the proportion of trials in which they produced gestures accurately in the delayed posttests. Language and communication abilities, fine motor proficiencies, and attention skills were found to be non-significant. We further established that gestural recognition would influence participants’ ability to produce gestures accurately, with this relation being partially mediated by the spontaneous imitation that occurred during training. These findings suggest that participants with ASD who better understand the meanings of gestures are more likely to spontaneously imitate the gestures demonstrated by the robot, which results in an enhancement in gestural production skills. These results are in line with previous research, which has shown that children with ASD have stronger motivations to imitate the gestures they understand [31]. This motivation may be reflected in the spontaneity in gestural imitation in the present study. Further intervention studies on the use of intransitive gestures should consider teaching children with ASD gestural recognition, followed by production (see [70, 72]).

## Conclusions

Our study is a pioneering work showing that robot-based intervention can prevent the delay in the production of intransitive gestures in young children with ASD. Theoretically, our findings have extended the previous research, which has posited that robot-based gestural training can be considered as an effective early intervention for gestural communication. It also has strong implications for the direction in which technology-based interventions for preschoolers with ASD should proceed. Practically, our research could promote the implementation of robot-based interventions in preschool education, even for children whose language and motor skills are delayed. Note that the language and communication ages of the participants with ASD participating in this study fell behind their chronological ages, suggesting that they might have language delay. Their fine motor proficiencies were poorer than those of the age-matched participants with typical development too. However, after four robot-based gestural training sessions, they could still produce gestures as accurately as the participants with typical development. Our protocol may also be useful in promoting general social competence, such as joint attention, perspective taking, and understanding others’ intentions.

That said, this study has a few limitations. First, there is no evidence showing that participants with ASD who received training actually applied the acquired gestural production skills when interacting with others in their daily lives. Hence, we cannot comment on the social utility of our intervention program. In future studies, we will observe the gestural communication of the participants in schools and at home for a longer period of time. Measuring generalization and long-term maintenance effects is always challenging, because it is difficult to maintain follow-up and control confounding variables. Therefore, there are very few long-term follow-up studies [46]. Besides, one may question whether or not the robot is better than humans at being a teacher for children with ASD, which was not addressed in the present study. We are currently conducting a new study, which compares the effectiveness of robot-based intervention to that of human-based intervention on conversation skills in children with ASD. We are not proposing that either humans or robots should be the teaching agent. Rather, we propose that robots can serve as an effective agent in teaching children with ASD social and communication skills. Related to the aforementioned limitation, the present study deployed NAO, a humanoid robot, as a teacher to train children with ASD gestural communication skills. However, a recent study has reported that there may be individual variations in the preferences for different types of robots for individuals with ASD [40]. Robins et al. [61] have even shown that children with ASD were more responsive toward a theatrical robot, which has plain appearance, than a humanoid robot. Future research should be cautious with the choice of robots.

## Abbreviations

AAC: Alternative and augmentative communication
ADI-R: Autism diagnosis interview-revised
ADOS: Autism diagnostic observation schedule
ANT: Attention network test
APA: American Psychiatric Association
ASD: Autism spectrum disorder
BOTTM-2: Bruininks-Oseretsky Test of Motor Proficiency, Second Edition
DOF: Degree of freedom
DS: Down’s syndrome
DSM-V: Diagnostic and Statistical Manual of Mental Disorders, Fifth Edition
MNS: Mirror neuron system
PECS: Picture Exchange Communication System
PEP-3: Psychoeducational Profile, Third Edition
SCQ: Social Communication Questionnaire
SGDs: Speech-generating devices
